# Dabigatran Acylglucuronide, the Major Metabolite of Dabigatran, Shows a Weaker Anticoagulant Effect than Dabigatran

**DOI:** 10.3390/pharmaceutics14020257

**Published:** 2022-01-22

**Authors:** Jong-Min Kim, Jihyeon Noh, Jin-Woo Park, Hyewon Chung, Kyoung-Ah Kim, Seung Bin Park, Jun-Seok Lee, Ji-Young Park

**Affiliations:** 1 Department of Clinical Pharmacology and Toxicology, Korea University College of Medicine, Korea University Anam Hospital, Seoul 02841, Korea; jmk157@korea.ac.kr (J.-M.K.); njh2535@korea.ac.kr (J.N.); kakim920@kumc.or.kr (K.-A.K.); 2 Department of Neurology, Korea University College of Medicine, Korea University Anam Hospital, Seoul 02841, Korea; parkzinu@korea.ac.kr; 3 Department of Clinical Pharmacology and Toxicology, Korea University Guro Hospital, Seoul 08308, Korea; hyewonchung@korea.ac.kr; 4 Department of Pharmacology, Korea University College of Medicine, Korea University, Seoul 02841, Korea; 2018010562@korea.ac.kr

**Keywords:** dabigatran, dabigatran acylglucuronide, thrombin generation, anticoagulation

## Abstract

Dabigatran (DAB) is an orally administered thrombin inhibitor. Both DAB and its main metabolite dabigatran acylglucuronide (DABG) have established anticoagulant effects. Here, we aimed to compare the relative anticoagulant effects of DABG and DAB in humans. Anticoagulant effects of DAB and DABG were measured in vitro using a thrombin generation assay. Additionally, their effects on other coagulation assays including PT, aPTT, TT, and fibrinogen were compared. Both DAB and DABG showed inhibitory effects on thrombin generation in a dose-dependent manner, but DABG exhibited a weaker inhibitory effect than that of DAB. The IC_50_ values of DAB and DABG on thrombin generation AUC were 134.1 ng/mL and 281.9 ng/mL, respectively. DABG also exhibited weaker anticoagulant effects than DAB on PT, aPTT, and TT. The results of the present study indicate that the anticoagulant effect of DABG, a main active DAB metabolite, is weaker than that of DAB.

## 1. Introduction

Thrombin is a plasma serine protease that plays a central role in coagulation and hemostasis and is produced by the proteolytic cleavage of prothrombin. Thrombin catalyzes the conversion of fibrinogen into fibrin, leading to thrombus formation [1].

Dabigatran (DAB) is a new, competitive, direct thrombin inhibitor widely used to treat and prevent thrombus formation and pulmonary embolism and to reduce the risk of stroke and systemic embolism [2,3]. Unlike heparin, which can only bind free thrombin, DAB is capable of binding and inhibiting both free and clot-bound thrombin [4].

As an ester prodrug, dabigatran etexilate (DABE) undergoes two sequential activation steps to form its pharmacologically active metabolite, DAB. After absorption, DABE is first metabolized to its intermediate metabolite, dabigatran ethyl ester (M2) by carboxylesterase 2 (CES2) in the intestine, and M2 is further converted to the final active metabolite DAB by carboxylesterase 1 (CES1) in the liver [5,6]. DAB is further metabolized to dabigatran acylglucuronide (DABG) by uridine 5-diphospho (UDP)-glucuronosyltransferase (UGT) in the liver. Glucuronidation of the carboxylate moiety is the major human metabolic pathway of DAB, and three UGTs, namely, *UGT1A9*, *UGT2B7*, and *UGT2B15* are involved. *UGT2B15* is the major isoform involved in the glucuronidation of DAB. It has been considered that drug monitoring is not routinely required for direct oral anticoagulants (DOACs), including dabigatran, due to predictable pharmacokinetics [7]. However, fast and accurate tests are needed to determine whether the patient has blood levels within therapeutic ranges of DOACs. Information about the anticoagulant status is required for optimal therapy, especially in case of intracranial bleeding or emergency interventions with potential risk of bleeding [7,8]. Routine global coagulation tests used to monitor DAB effects are PT (prothrombin time), expressed as the international normalized ratio (INR), aPTT (activated partial thromboplastin time) or TT (thrombin time). However, these tests were employed for assessment of the pharmacological effect of DAB, the relationship between these coagulation assays and DAB; blood levels showed inadequate sensitivity to support routine use either for excluding the presence of DAB or for determining DAB concentration [9].

Previously, it has been reported that approximately 20% of DAB is conjugated to form DABG [1,10,11] (Table 1). It is known that DABG is pharmacologically fully active and that the anticoagulant effect of DABG is comparable to that of DAB [12]. Therefore, glucuronidation is believed not to affect the clinical efficacy of DAB since the sum of concentrations of DAB and DABG is not changed [1].

Conversely, due to the relatively lower DABG concentration compared to the DAB concentration, the contribution of DABG to the anticoagulant effect is considered to be minor [1]. However, it has been recently reported that the plasma concentration of DABG is higher than that of DAB [13,14], suggesting that DABG could play a more crucial role than has been thought [1]; systemic exposure of DABG is 2.4-fold greater and *C_max_* (peak plasma concentration) of DABG is 3.1-fold higher than that of DAB after a single oral dose of 150 mg DABE in healthy subjects [13] (Table 1). The discrepancy arises from different sample extraction methods in the analysis step [13,14]. These results suggest that due to inaccurate measurements of DAB and DABG concentrations, the relationship with the various coagulation tests with DAB treatment could reveal poor results [9].

In this study, we hypothesized that DAB and DABG could have different anticoagulant effects; thus, we compared the relative anticoagulant effects of DAB and DABG using coagulation assays at therapeutic blood levels.

## 2. Materials and Methods

### 2.1. Reagents

DAB and DABG were purchased from TRC Canada (Toronto, ON, Canada). Synthetic phospholipids (PL) were obtained from Avanti Polar Lipids Inc. (Alabaster, AL, USA) and used as vesicles consisting of phosphatidylserine, phosphatidylethanolamine, and phosphatidylcholine (1:1:3, mmol:mmol:mmol). Recombinant tissue factor (TF) (Hemoliance^®^ RecombiPlasTin) was obtained from the Instrumentation Laboratory (Milan, Italy) [16]. The fluorogenic substrate Z-Gly-Gly-Arg-aminomethyl coumarin (ZGGR-AMC) was purchased from Bachem Co. (Basel, Switzerland). Other chemicals were of analytical grade or higher purity and were obtained from commercial suppliers. The concentrations of DAB and DABG used in this study ranged from 0 to 1000 ng/mL (0–2.12 μM for DAB and 0–1.54 μM for DABG) based on the clinically observed data [13,14]. The stock solution (1 mg/mL) was diluted with plasma consecutively, and DAB and/or DABG-spiked plasma samples were prepared. All spiked samples were prepared 20 min before the experiments and kept at 37 °C.

### 2.2. Sample Collection

The samples were collected following the Declaration of Helsinki and the collection was approved by the Ethics Committee of Anam Hospital, Korea University, Seoul, Korea.

Venous blood was collected from healthy, fasting, drug-free donors (aged 20–55 years) by venipuncture of a peripheral vein into conventional 3.2% (109 mM) sodium citrate anticoagulated blood tubes (9:1 *v*/*v*, Becton Dickinson, NJ, USA). Plasma was isolated by double centrifugation at 1500× *g* for 15 min at 4 °C, aliquoted, and stored at −80 °C until testing. Frozen plasma sample aliquots were thawed and heated to 37 °C for 5–10 min before the experiments, and unfrozen plasma samples were centrifuged at 14,000× *g* for 5 min before use.

### 2.3. Coagulation Assays (TT, PT, aPTT, and Fibrinogen)

The plasma samples were loaded in STA^®^ Compact Max (Stago, Asnières, France) using the same batch of reagents. To measure the TT, a plasma sample (100 μL) and STA^®^-thrombin were mixed in a cuvette. To measure the PT, STA^®^ Neoplastine^®^ CI reagent (100 μL) was added to a cuvette with a plasma sample (50 μL). To measure the aPTT, 50 μL of plasma sample was required, which was placed in a cuvette in the presence of STA^®^-PTT (50 μL) and CaCl_2_ 0.025 M (50 μL). In the case of the fibrinogen measurement, the plasma sample (7.5 μL), which was automatically diluted with Owren–Koller buffer (142.5 μL), was placed into a cuvette and then STA^®^-LIQID FIB (50 μL) was added. Calibrators and internal controls were used according to the manufacturer’s instructions. Measurements were performed in duplicate for each experiment.

### 2.4. Thrombin Generation Assay

Thrombin generation was determined in citrated plasma using a commercially available assay kit (Technothrombin^®^ TGA; Technoclone, Vienna, Austria) on a fully automated, computer-controlled microplate reader (Flexstation 3; Molecular Devices, San Jose, CA, USA) with a 360 nm excitation wavelength and 460 nm emission wavelength, using a specially adapted software (Technothrombin TGA, Technoclone, Vienna, Austria) [17,18]. Plasma samples (30 μL) were loaded onto 96-well plates and then mixed with 25 μL of 400 μmol/L fluorogenic substrate (Z-Gly-Gly-Arg-AMC; Bachem, Bubendorf, Switzerland). The assay was initiated by dispensing 10 μL of recombinant human tissue factor (final concentration 5 pmol/L) lipidated with synthetic phospholipids and 10 μL of CaCl_2_ (final concentration 15 mmol/L). Measurements were performed in duplicate for each experiment. Thrombin generation was measured and compared in terms of peak thrombin concentration (*C_max_*), endogenous thrombin potential (AUC), time to *C_max_* (*T_max_*), and lag time.

### 2.5. Docking Simulation

The binding mode between DAB and DABG was evaluated based on AutoDock Vina docking simulations (version 1.1.2) [19]. The protein crystal structure was obtained from the PDB database (PDB code: 1KTS), consisting of thrombin and an ester-protected form of dabigatran at 2.4 Å resolution. The initial structures of ligands for the docking simulation were prepared via geometry optimization using an empirical force field, MM2. For each ligand docking simulation, the top 10 conformations of ligands were used to evaluate the binding modes with PyMOL (v. 2.3.5).

### 2.6. Statistical Analysis

All data are expressed as mean ± standard deviation (SD). Regression analysis was used to estimate IC_50_ values from the inhibition plot. Differences between groups were analyzed by one-way analysis of variance (ANOVA) followed by Bonferroni’s test or Student’s *t*-test, where appropriate. Statistical analyses were performed using SAS^®^ 9.4 (SAS Institute Inc., Cary, NC, USA) and Microsoft Excel 2019 (Microsoft Corp., Redmond, WA, USA). Statistical significance was set at *p* < 0.05.

## 3. Results

### 3.1. Effect of DAB and DABG on Thrombin Generation Assay

**Figure 1:**
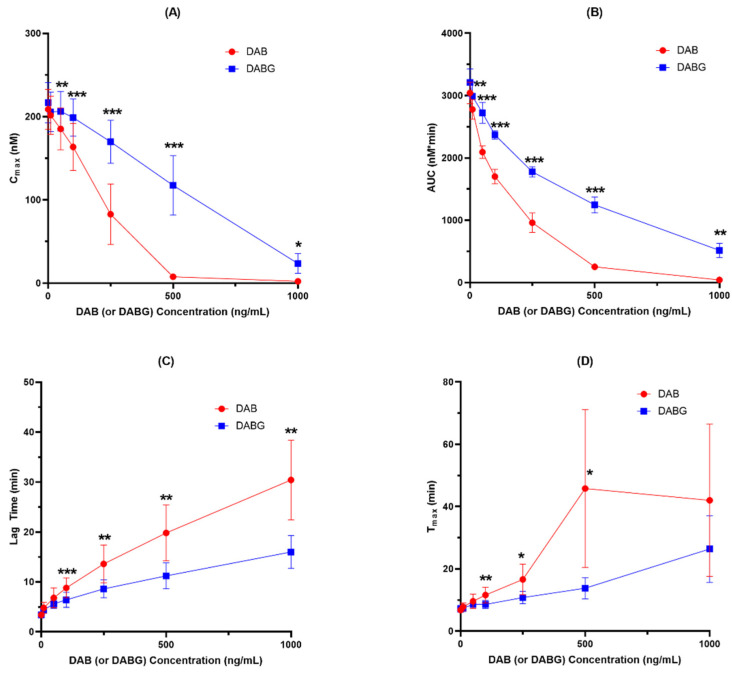
Inhibition of thrombin generation by DAB and DABG (*n* = 5). (**A**) *C_max_*; (**B**) AUC; (**C**) Lag time and (**D**) *T_max_*. *, *p* < 0.05; **, *p* < 0.01; ***, *p* < 0.001.

The inhibitory effects of DAB and DABG on thrombin generation are shown in Figure 1 and Figure S1.

**Figure 2:**
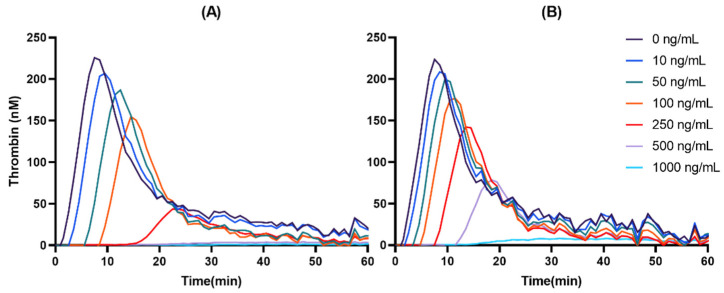
Representative thrombin-generation inhibition by DAB (**A**) and DABG (**B**).

Both DAB and DABG inhibited thrombin generation in a concentration-dependent manner. In the *C_max_* and AUC measurements, DAB inhibited thrombin generation more than DABG. Likewise, lag time and *T_max_* showed more prolongation for DAB than for DABG. The kinetics of thrombin generation by DAB and DABG are shown in Figure 2.

Comparing the inhibitory potency of DAB and DABG on thrombin generation, the calculated IC_50_ values of DAB and DABG based on AUCs were 134.1 ng/mL and 281.9 ng/mL, respectively, suggesting that DAB showed 2.10-fold more potent inhibitory effect than DABG. The calculated IC_50_ values of DAB and DABG for thrombin generation are shown in Table 2.

### 3.2. Anticoagulant Effects of DAB and DABG

**Figure 3:**
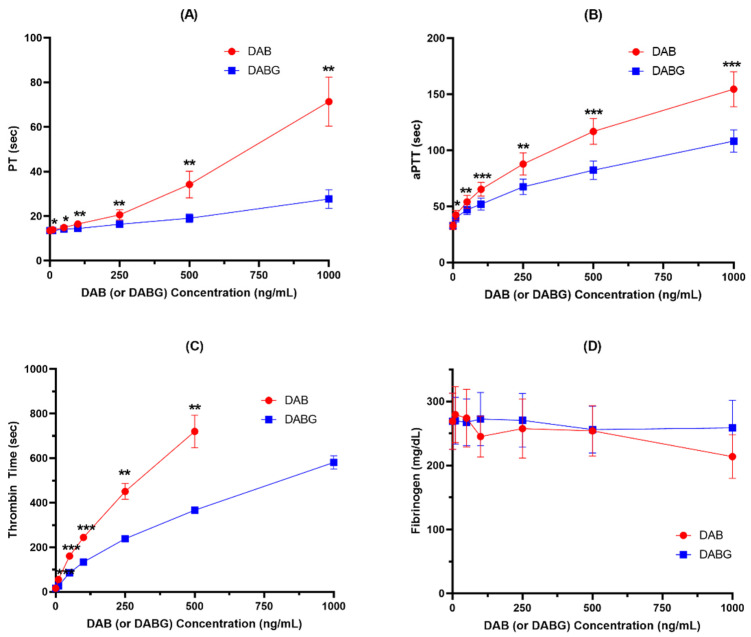
Anticoagulant effects of DAB and DABG assessed by PT (**A**), aPTT (**B**), TT (**C**), and fibrinogen (**D**) (*n* = 5). *, *p* < 0.05; **, *p* < 0.01; ***, *p* < 0.001.

In addition to thrombin generation assay, the anticoagulant effects of DAB and DABG on coagulation systems, including PT, aPTT, TT, and fibrinogen, were compared and are shown in Figure 3 and Figure S2.

Both DAB and DABG produced inhibitory effects on PT, aPTT, and TT in a concentration-dependent manner, but not on fibrinogen. Consistent with the results observed for thrombin generation, DAB exhibited a stronger anticoagulant effect than DABG. However, neither DAB nor DABG had an inhibitory effect on fibrinogen formation.

### 3.3. Combination Effect of DAB and DABG on Thrombin Generation

DABG is the main active metabolite formed from DAB, and both are found simultaneously in the plasma after administration in humans. Thus, we assessed the combined effect of DAB and DABG, using a thrombin generation assay to determine whether DAB and DABG exhibited additive effects. Considering that DABG showed a relatively weaker anticoagulant effect than DAB, the combination of DAB and DABG could present stronger anticoagulant effects than DAB or DABG alone. 

**Figure 4:**
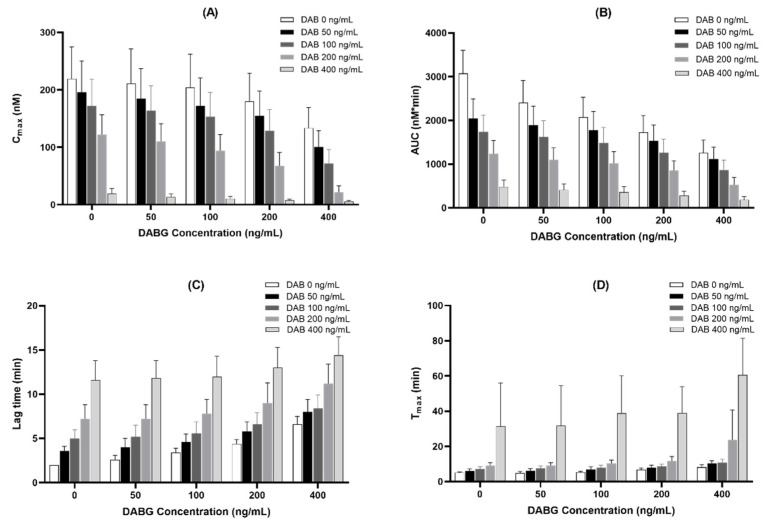
Additive inhibitory effect of co-treatment with DAB and DABG on thrombin-generation assay (*n* = 5). (**A**), *C_max_*; (**B**), AUC; (**C**), lag time; (**D**), *T_max_*.

When the mixture of DAB and DABG was co-incubated, the inhibitory effect of thrombin generation was weaker than that of DAB alone, considering the same amount, but was stronger than DABG alone, indicating that DAB and DABG showed an additive effect on their anticoagulant effect and that the anticoagulant effect of DABG is not equal to that of DAB (Figure 4).

## 4. Discussion

It is well known that DABG is the major active metabolite, and its anticoagulant effect is comparable to that of DAB [12]. Its anticoagulant effect corresponding to its level in the blood (i.e., pharmacokinetic and pharmacodynamic interaction) was assessed by calculating the total DAB levels (i.e., a summation of blood levels of DAB and DABG after glucuronic cleavage) [1,20,21,22,23]. However, we found that the anticoagulation effect of DABG was not equal to that of DAB; it is far weaker than that of DAB. When we calculated the IC_50_ value for *C_max_* of thrombin generation assay, DAB showed 185.9 ng/mL (0.394 μM), whereas that of DABG was 470.3 ng/mL (0.726 μM), indicating an 84% weaker effect of DABG. Furthermore, DABG exhibited a 52% weaker inhibitory effect than DAB when the AUC of thrombin generation was compared. 

Ebner et al. reported that DAB and DABG had a similar anticoagulant effect: the anticoagulant activities of DAB and DABG were compared using the assessment of the concentrations required for a doubling aPTT and their concentrations for DAB and DABG were 0.45 μM and 0.46 μM, respectively [12]. However, when we compared the doubling time for aPTT in our study, the observed concentration was 0.38 μM for DAB and 0.64 μM for DABG, DABG showing a 40% weaker effect compared to DAB. Consistently, DABG showed a weaker anticoagulant effect than DAB in other coagulant assays including PT and TT. 

**Figure 5:**
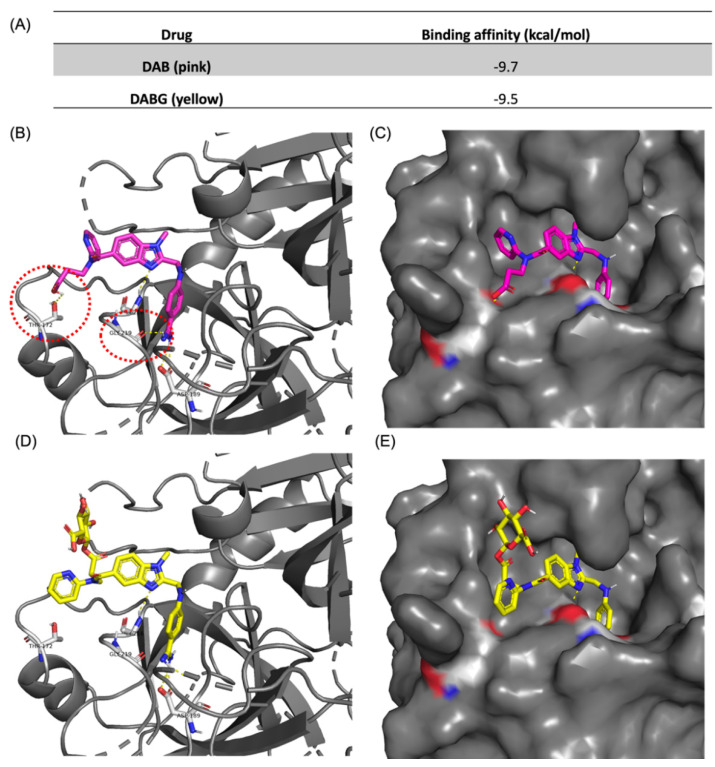
Docking-simulation results between thrombin and ligands. (**A**) Binding affinity energies obtained from the top-hit docking mode of DAB and DABG using Autodock Vina. Docking conformation of DAB with thrombin with (**B**) cartoon plot and (**C**) protein surface plot. Docking conformation of DABG with thrombin with (**D**) cartoon plot and (**E**) protein surface plot. Docking results and hydrogen bonding were visualized using PyMOL (v2.3.5).

Additionally, molecular docking results showed distinctive affinities of DAB and DABG to thrombin, −9.7 kcal/mol for DAB and −9.5 kcal/mol for DABG, respectively, (Figure 5A). Though we did not measure binding affinity experimentally in vitro, in silico binding energy of DAB is equivalent to a dissociation constant (K_d_) value of 76.8 nM and that of DABG corresponds to 107.8 nM, which shows that those two ligands reveal significantly distinctive affinity. Based on the docking conformations, we observed that both DAB and DABG bind to the previously reported X-ray crystal (PDB: 1KTS) identified pocket site, and the docking conformation of both compounds shared the binding mode for the core benzimidazole ring and guanidinium motifs (Figure 5B–E). However, the pyridine ring conformation was flipped in the case of DABG, since acyl-glucuronide prefers to reside at the site where pyridine was placed, in the case of DAB. More importantly, this conformational flip in DABG disrupts two hydrogen-bonding interactions between the ligand and thrombin at threonine 172 (Thr172) and glycine 219 (Gly219), resulting in a lower binding affinity (Figure 5B,D). Taken together with the binding energy and mode, it is apparent that DABG and DAB exhibit distinctive binding to thrombin, based on in silico simulation results.

We cannot give the exact reason(s) why DABG showed a weaker anticoagulant effect than DAB in our study, in contrast to the previous results. Interestingly, a previous study reported that both DAB and DABG showed a similar anticoagulant effect in aPTT at concentrations lower than 1 μM, but exhibited a difference in doubling time for aPTT at concentrations higher than 1 μM. When we reviewed the literature, we found that the experiments differed in the citrate concentration of the plasma samples used (conventional citrate tube [10.9 mM] vs. 10.6 mM for the previous experiment) [12]. It is well known that sodium citrate concentration is the main factor affecting coagulation assay results [24,25,26]. According to the literature, comparing the effect of anticoagulant drugs at 3.2% (109 mmol/L) and 3.8% (129 mmol/L) citrate concentrations showed that effects at a low concentration of 3.2% citrate were not as pronounced as those at 3.8% citrate [25,27]. However, this may not explain the different results in our study, and further evaluation is needed to elucidate the discrepancy.

The blood levels of DABG are known, or believed to be, maintained at a relatively low concentration, at approximately 20% of DAB levels [1,11]. Therefore, considering the relatively lower blood levels of DABG, our results suggest that the anticoagulant effect of DABG may play a minor role compared to that of DAB. However, according to recent literature, the plasma levels of DABG are maintained at a higher level than those of DAB [13,14]. After a single-dose administration of 150 mg DABE, the observed average peak plasma concentration of DAB was 87 ng/mL, whereas that of DABG was 266.8 ng/mL (i.e., 3-fold higher). These *C_max_* values were approximately 47% for DAB and 57% for DABG for the respective IC_50_ values. When we assessed the effects of mixed DAB/DABG in the thrombin generation assay, we observed an additive effect. For instance, mixed DAB 100 ng/mL with DABG 100 ng/mL exhibited the inhibition of 51.7% for *C_max_* and 30.2% for AUC in the thrombin generation assay in this study. However, assuming that the antithrombotic effect of DABG is equal to that of DAB, as believed previously, its inhibition was calculated as 60.0% for *C_max_* and 41.3% for AUC in thrombin generation assay at these concentrations. These results suggest that the previously estimated anticoagulant effect of DAB may have been exaggerated. 

DABG is formed from DAB by glucuronidation. and *UGT2B15* is the major isoform involved in the DAB glucuronidation [12]. *UGT2B15* genetic differences have been shown to impact *UGT2B15*-mediated glucuronidation ability for some substrates [28]. Especially, UGT2B15*2, which is the highly prevalent, non-synonymous, single nucleotide polymorphism (SNP) present in approximately 50% of Caucasians, g.253G>T (D85Y; rs1902023), is a significant determinant of *UGT2B15* interindividual variation [29,30]. Because it is considered that DABG has a pharmacological effect comparable to DAB, the metabolic activity of *UGT2B15* could be neglected. However, our data indicate that the change in metabolic disposition of *UGT2B15*, followed by its genetic polymorphisms or drug interaction, could influence the blood level of DAB and DABG in the body and affect the anticoagulant effect. Therefore, considering that the potential effect of *UGT2B15* polymorphisms [29,30], *UGT2B15* poor metabolizer (e.g., UGT2B15*2/*2) may exhibit a more potent anticoagulant effect than *UGT2B15* extensive metabolizer by higher exposure of DAB than its extensive metabolizer.

Due to technical issues in the measurement of DAB, DABG and commercial availability, the blood levels of DABG were determined using an indirect method of converting DABG to DAB by an alkali cleavage instead of direct measurement [13]. Additionally, it was thought that DABG plays a minor role in the anticoagulant effect of DAB due to its relatively low concentrations. However, considering the relatively higher blood levels but the weaker anticoagulant effect of DABG than those of DAB in this study, the anticoagulant effects of both DAB and DABG should be taken into consideration. A discrepancy between global coagulation testing and DAB plasma concentration was shown in a considerable proportion of patients, depending on the test platforms and reagent, with values ranging from 6% to 62% [31]. Misinterpretation might endanger patients treated with DAB. Inaccurate measurements of DAB and DABG concentrations are a factor causing poor results. In addition, we believe that the difference in anticoagulant effects between DAB and DABG is also considered problematic.

## 5. Conclusions

The results of the present study indicate that the anticoagulant effect of DABG, a main active metabolite of DAB, is weaker compared to that of DAB. Current experiment evidence supports that poor relationship between DAB concentrations and coagulation testing could be attributed to difference in anticoagulant effect of DAB and DABG.

## Figures and Tables

**Table 1:** Summary of the pharmacokinetic profiles of DAB and DABG after oral administration of 150 mg DABE.

Compound	*C_max_* (ng/mL)	*T_max_* (h)	Half Life (h)	*AUC_inf_* (ng × h/mL)	Reference
Total DAB	145	2.0	8.9	1261	[10]
DAB	122	2.0	8.7	1079	
DABG *	23	-		182	
DAB + DABG	111	2.0	8.7	904	[15]
DAB	87	2.0	6.3	690	[13]
DABG	267	2.0	6.4	1642	

*C_max_*, maximum plasma concentration; *T_max_*, time to reach *C_max_*; *AUC_inf_*, total area under the plasma concentration-time curve (AUC) form 0 h to infinity; *, the value of DABG is calculated as total DAB-DAB.

**Table 2:** IC_50_ values of dabigatran (DAB) and dabigatran acylglucuronide (DABG) on thrombin generation assays (*n* = 5).

Parameter	DAB	DABG	Ratio (Molar Ratio *)
IC_50_ for *C_max_*	185.9 ± 40.3 ng/mL (393.9 nM)	470.3 ± 87.8 ng/mL (726.2 nM)	2.53 (1.84)
IC_50_ for *AUC*	134.1 ± 31.5 ng/mL (284.1 nM)	281.9 ± 48.2 ng/mL (435.3 nM)	2.10 (1.52)

Data are expressed mean ± SD; *, the ratio of DABG to DAB.

## Data Availability

The data presented in this study are available on request from the corresponding author.

## Supplementary Materials

The following supporting information can be downloaded at: https://www.mdpi.com/article/10.3390/pharmaceutics14020257/s1, Figure S1: Inhibition of thrombin generation by DAB and DABG (calculated as molar concentration) (*n* = 5). ((A), *C_max_*; (B), *AUC*; (C), Lag time; (D), *T_max_*). Figure S2: Anticoagulant effects of DAB and DABG (calculated as molar concentration) assessed by PT (A), aPTT (B), TT (C), and fibrinogen (D).
