# Supplementary material for: Dabigatran Acylglucuronide, the Major Metabolite of Dabigatran, Shows a Weaker Anticoagulant Effect than Dabigatran
Source: Pharmaceutics. 2022 Jan 22;14(2):257. doi: 10.3390/pharmaceutics14020257 (PMC8875894; doi:10.3390/pharmaceutics14020257)
Supplement: Supplementary file 1 [file pharmaceutics-14-00257-s001.zip › pharmaceutics-1514907-supplementary.pdf]

pharmaceutics

MDPI

Supplementary Materials: Dabigatran Acylglucuronide, the Major Metabolite of Dabigatran, Shows a Weaker Anticoagulant Effect than Dabigatran

Jong-Min Kim, Jihyeon Noh, Jin-Woo Park, Hyewon Chung, Kyoung-Ah Kim, Seung Bin Park, Jun-Seok Lee and Ji-Young Park

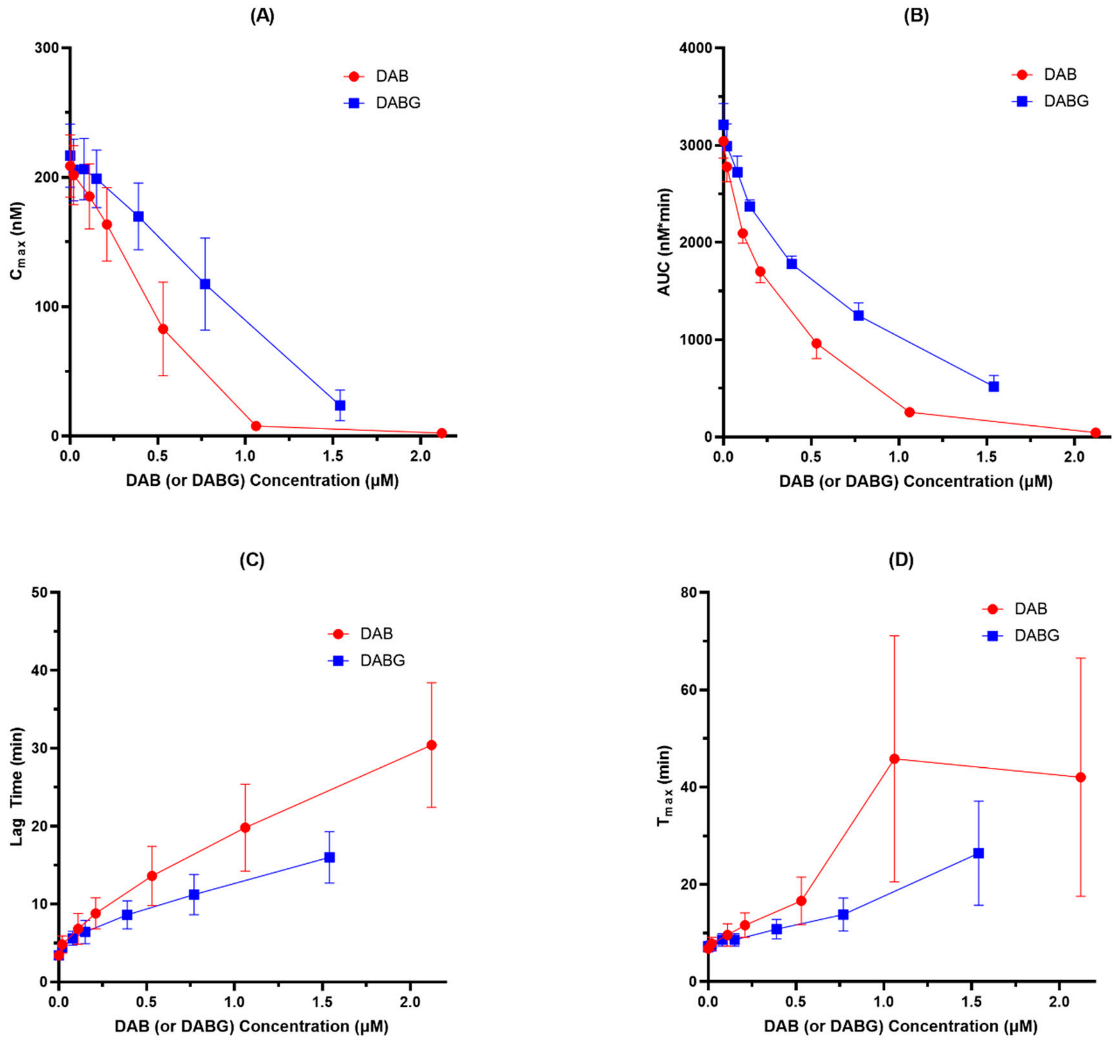

Figure S1. Inhibition of thrombin generation by DAB and DABG (calculated as molar concentration) (n = 5). (**A**) C_{max}; (**B**) *AUC*; (**C**) Lag time and (**D**) T_{max}.

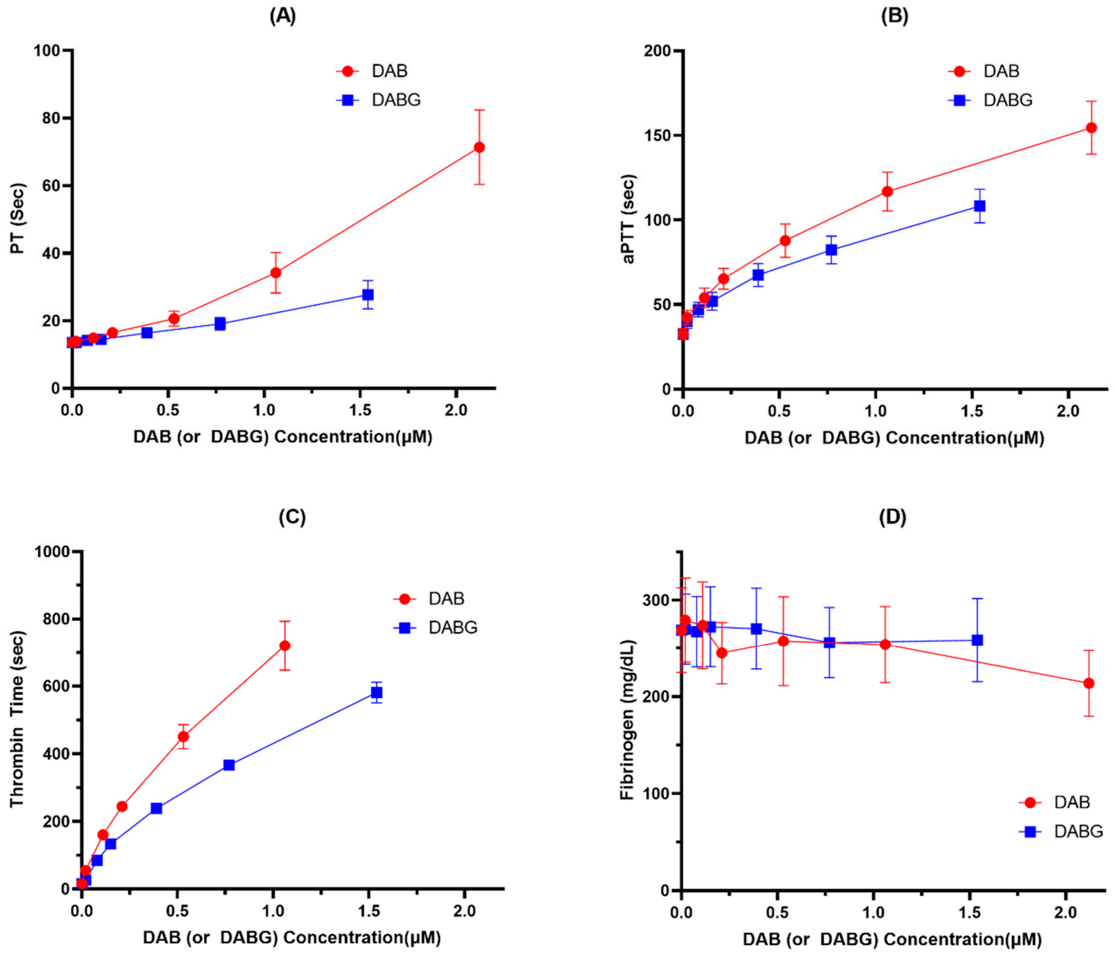

Figure S2. Anticoagulant effects of DAB and DABG (calculated as molar concentration) assessed by (**A**) PT, (**B**) aPTT, (**C**) TT, and (**D**) fibrinogen.
